# The H-NS Regulator Plays a Role in the Stress Induced by Carbapenemase Expression in Acinetobacter baumannii

**DOI:** 10.1128/mSphere.00793-20

**Published:** 2020-08-26

**Authors:** Fanny Huang, Noelle Fitchett, Chelsea Razo-Gutierrez, Casin Le, Jasmine Martinez, Grace Ra, Carolina Lopez, Lisandro J. Gonzalez, Rodrigo Sieira, Alejandro J. Vila, Robert A. Bonomo, Maria Soledad Ramirez

**Affiliations:** a Center for Applied Biotechnology Studies, Department of Biological Science, College of Natural Sciences and Mathematics, California State University, Fullerton, Fullerton, California, USA; b Instituto de Biología Molecular y Celular de Rosario (IBR, CONICET-UNR), Rosario, Argentina; c Área Biofísica, Facultad de Ciencias Bioquímicas y Farmacéuticas, Universidad Nacional de Rosario, Rosario, Argentina; d Fundación Instituto Leloir—IIBBA CONICET, Buenos Aires, Argentina; e Research Service and GRECC, Louis Stokes Cleveland Department of Veterans Affairs Medical Center, Cleveland, Ohio, USA; f Departments of Medicine, Pharmacology, Molecular Biology and Microbiology, Biochemistry, Proteomics and Bioinformatics, Case Western Reserve University School of Medicine, Cleveland, Ohio, USA; g CWRU-Cleveland VAMC Center for Antimicrobial Resistance and Epidemiology (Case VA CARES), Cleveland, Ohio, USA; Escola Paulista de Medicina/Universidade Federal de São Paulo

**Keywords:** *Acinetobacter baumannii*, H-NS, stress, carbapenemases, *Acinetobacter*, carbapenems

## Abstract

Disruption of the histone-like nucleoid structuring protein (H-NS) was shown to affect the ability of Gram-negative bacteria to regulate genes associated with virulence, persistence, stress response, quorum sensing, biosynthesis pathways, and cell adhesion. Here, we used the expression of metallo-β-lactamases (MBLs), known to elicit envelope stress by the accumulation of toxic precursors in the periplasm, to interrogate the role of H-NS in Acinetobacter baumannii, together with other stressors. Using a multidrug-resistant A. baumannii strain, we observed that H-NS plays a role in alleviating the stress triggered by MBL toxic precursors and counteracts the effect of DNA-damaging agents, supporting its role in stress response.

**IMPORTANCE** Carbapenem-resistant A. baumannii (CRAB) is recognized as one of the most threatening Gram-negative bacilli. H-NS is known to play a role in controlling the transcription of a variety of different genes, including those associated with the stress response, persistence, and virulence. In the present work, we uncovered a link between the role of H-NS in the A. baumannii stress response and its relationship with the envelope stress response and resistance to DNA-damaging agents. Overall, we posit a new role of H-NS, showing that H-NS serves to endure envelope stress and could also be a mechanism that alleviates the stress induced by MBL expression in A. baumannii. This could be an evolutionary advantage to further resist the action of carbapenems.

## Abstract (precis)

Carbapenem-resistant A. baumannii (CRAB) is recognized as one of the most threatening Gram-negative bacilli. H-NS is known to play a role in controlling the transcription of a variety of different genes, including those associated with the stress response, persistence, and virulence. In the present work, we uncovered a link between the role of H-NS in the A. baumannii stress response and its relationship with the envelope stress response and resistance to DNA-damaging agents. Overall, we posit a new role of H-NS, showing that H-NS serves to endure envelope stress and could also be a mechanism that alleviates the stress induced by MBL expression in A. baumannii. This could be an evolutionary advantage to further resist the action of carbapenems.

## OBSERVATION

Acinetobacter baumannii is a nosocomial pathogen, frequently resistant to multiple drugs, that causes a wide variety of infections associated with high mortality rates. Carbapenem-resistant A. baumannii (CRAB) has frequently been reported among hospital patients (1). In addition, the CDC’s 2019 Antibiotic Resistance Threats Report moved CRAB into the urgent-threats category (2). The expression of carbapenemases is critical for this organism to thrive under the selection pressure of these antibiotics. Under permissive conditions (the absence of antibiotics), the expression of some metal-dependent carbapenemases compromises the fitness of A. baumannii. Production of metallo-β-lactamases (MBLs) in uncommon hosts triggers different responses associated with envelope stress, such as activation of the periplasmic DegP homeostatic system and enhancement of outer membrane vesicle production to relieve the stress generated (3). Despite the increased knowledge gained in recent years regarding the epidemiology, pathogenicity, and antimicrobial resistance of A. baumannii (2, 4), the response of this pathogen to stressful environments is still not completely understood.

H-NS is a histone-like nucleoid structuring protein that serves as a global repressor. H-NS has been shown to be involved in the stress response in Gram-negative bacilli, such as Vibrio cholerae and Escherichia coli (5, 6). H-NS is known to protect bacteria from environmental stresses through regulation of the transcription and translation of virulence genes, quorum osmolarity, stress, etc. (7, 8).

In A. baumannii, the disruption of H-NS was found to affect the ability of the bacterium to regulate genes associated with persistence and virulence (9). However, the role of H-NS in the stress response in A. baumannii has not been addressed yet. Here, we aimed to test the role of H-NS in the A. baumannii stress response and to discover how this could be linked with the success of multidrug-resistant A. baumannii in the hospital environment. Recent studies have shown that the production of MBLs exerts an envelope stress in an A. baumannii laboratory strain, resulting in growth defects (3). In this way, to study the role of H-NS in overcoming different kinds of stress, we utilized and evaluated the expression of three MBLs—NDM-1, VIM-2, and SPM-1—as stressors in the periplasmic space of strain AB5075, as well as different known DNA-damaging agents.

Lopez et al. have shown that inefficient processing upon translocation of nonfrequent carbapenemases in A. baumannii, such as VIM-2 and SPM-1, compromises bacterial fitness by triggering an envelope stress (3). In contrast, expression of NDM-1 (a common resistance determinant in A. baumannii) is coupled to efficient processing, without causing any stress (3). In this way, this system represents a unique model for testing the envelope stress response, since this stress can be regulated by varying the expression levels of MBLs, which directly affect the accumulation of toxic precursors in the periplasmic space.

**FIG 1:**
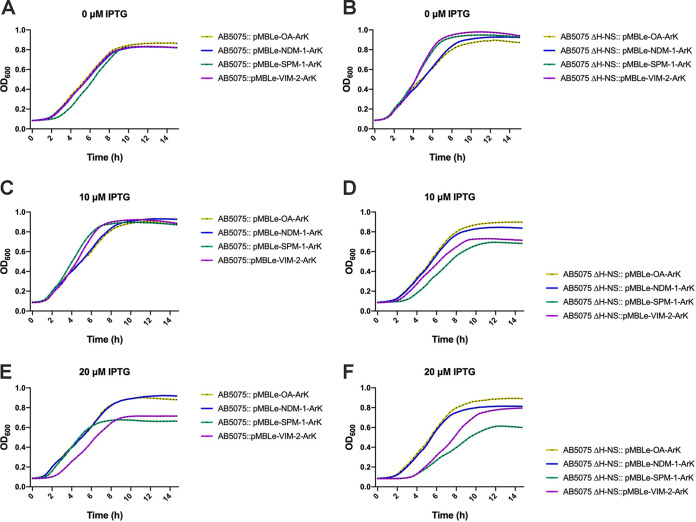
Growth curves of strains AB5075 and AB5075 Δ-*hns* either carrying the empty vector (pMBLe-OA) or expressing *bla*_NDM-1_, *bla*_VIM-2_, or *bla*_SPM-1_. Strains AB5075 and AB5075 Δ-*hns* with pMBLe-OA-ArK, pMBLe-VIM-2-ArK, pMBLe-SPM-1-ArK, or pMBLe-NDM-2-ArK were grown in LB broth plus 0 (A and B), 10 (C and D), or 20 (E and F) μM IPTG. The OD_600_ of the cultures was recorded every 20 min for 15 h. The data presented are means from three independent experiments.

To study the possible role of H-NS in relieving envelope stress and overcoming the expression of NDM-1, VIM-2, and SPM-1, growth curves of AB5075 and AB5075 Δ-*hns* expressing the different MBLs were analyzed. The mutant strain did not show impaired growth either with the empty vector or when expressing NDM-1 relative to the wild-type strain (Fig. 1A to C). Instead, the expression of VIM-2 or SPM-1 affected the growth of AB5075, in line with previous studies (3). This effect was more pronounced in a Δ-*hns* background, indicating that a lack of H-NS impairs the growth of strains expressing SPM-1 and VIM-2 (Fig. 1B and C).

Growth curves were unaltered when MBL expression was not induced (Fig. 1A and B), suggesting that H-NS plays a role in managing the accumulation of toxic precursor forms of SPM-1 and VIM-2. Our results also showed that when SPM-1 and VIM-2 were produced in relatively small amounts (0 and 10 μM isopropyl-β-d-thiogalactopyranoside [IPTG]), A. baumannii was able to withstand much of the impact on growth (Fig. 1A to D). The effect of the fitness cost on the induction of SPM-1 and VIM-2 became evident at 20 μM IPTG (Fig. 1E and F).

**FIG 2:**
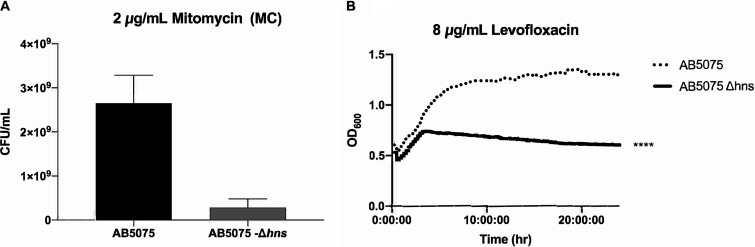
H-NS role in overcoming DNA damage. (A) Mitomycin C (MC) survival assay of strains AB5075 and AB5075 Δ-*hns*. The cells were grown in LB broth overnight and then serially diluted in agar plates containing MC at 0.2 μg/ml. The data presented are means ± standard deviations from three independent experiments. (B) Growth curves of A. baumannii strains AB5075 and AB5075-*Δhns* in LB broth supplemented with 8 μg/ml levofloxacin. Growth was recorded (OD_600_) over 24 h. Statistical analysis was performed using a Mann-Whitney test (*n* = 3; *P* < 0.05). The data presented are means from three independent experiments.

We next sought to evaluate whether H-NS is also involved in the ability of A. baumannii to overcome other stressors, such as the DNA-damaging agents mitomycin C (MC) and levofloxacin. AB5075 Δ-*hns* exhibited decreased viability when exposed to MC (Fig. 2A). Also, the bacterial growth curve in the presence of levofloxacin showed impaired growth for AB5075 Δ-*hns* (Fig. 2B). Overall, these data show that H-NS is involved in different A. baumannii stress responses.

The stress response in A. baumannii is linked to limitation of essential nutrients, antibiotic treatment, oxidative damage, and exposure to antiseptics, among other factors (10). When exposed to stress environments such as pleural fluid, A. baumannii can control the expression of different genes to overcome the stress and persist under the stressor signals (11).

In some Gram-negative bacilli, the role of H-NS in stress response has been well-characterized; e.g., in Vibrio cholerae, the deletion of *hns* has been shown to induce an envelope stress response causing increasing expression of *rpoE* and the regulators *rseA*, *rseB*, and *rseC*, suggesting a role for *hns* in cell envelope biogenesis (5). However, data on A. baumannii are scarce (9, 12).

Recent studies showed that periplasmic stress generated by the production of toxic MBLs can be alleviated by an increase in the production of outer membrane vesicles (hypervesiculation phenotype) enclosing non-host-adapted MBLs. Along with membrane vesiculation, the activation of periplasmic proteases also acts to relieve the accumulation of toxic MBLs in the periplasm in nonfrequent hosts (3). Here, we show that the H-NS regulator (used by the highly resistant and hypervirulent strain AB5075) also contributes to coping with the expression of MBLs. We observed that AB5075 expressed NDM-1 without growth defects. In contrast, the expression of VIM-2 and SPM-1 compromised A. baumannii survival, triggering a stress response that is H-NS dependent.

To further understand the epidemiology and host specificity of MBLs, we explored if low expression levels of VIM-2 and SPM-1 could confer carbapenem resistance without affecting bacterial fitness. We determined the imipenem (IMI) MICs of AB5075 and AB5075 Δ-*hns* expressing these MBLs at different levels. Interestingly, we observed that SPM-1 can confer resistance to IMI (see Fig. S1 in the supplemental material) under tight expression levels (0 μM IPTG). Upon the addition of 10 μM IPTG, instead, we observed IMI MIC values similar to those for the control strain lacking the lactamase gene (Fig. S1). In contrast, VIM-2 was not able to confer carbapenem resistance under any condition assayed. The impact of SPM-1 production by AB5075 might explain in part why A. baumannii strains carrying *bla*_SPM-1_ have rarely been reported, especially in Brazil, where the SPM-1-producing Pseudomonas aeruginosa ST277 clone is endemic. To date, only one case of an A. baumannii strain producing SPM-1 has been reported in that country (13), and such a result is open to question, since the identification at species level was performed only by a phenotypic automated system, and the *bla*_SPM-1_ gene was not sequenced. Finally, when the IMI MICs were determined in AB5075 Δ-*hns* expressing the different MBLs, an amplified effect of toxicity and a detrimental impact on the antibiotic resistance phenotype was observed (Fig. S1), in agreement with previous results. Overall, our results support the infrequent spread of SPM-1 and VIM-2 in A. baumannii and show that the impact of the expression levels on bacterial fitness is strongly dependent on each MBL, an observation that deserves further investigation.

10.1128/mSphere.00793-20.1 FIG S1 Imipenem MICs of strains AB5075 and AB5075 Δ-*hns*, carrying the empty vector (pMBLe-OA) or expressing *bla*_NDM-1_, *bla*_VIM-2_, or *bla*_SPM-1_ at 0 and 10 μM IPTG. Data correspond to five independent experiments and are shown as the mean values. Error bars represent standard deviations. Download FIG S1, PDF file, 0.04 MB. Copyright © 2020 Huang et al. 2020 Huang et al. This content is distributed under the terms of the Creative Commons Attribution 4.0 International license.

We also observed that H-NS is involved in the stress response, not only alleviating the stress imposed by expression of VIM-2 and SPM-1, but also that imposed by DNA-damaging agents.

Collectively, our observations suggest that H-NS serves to overcome envelope stress and could also be a possible mechanism that may allow alleviation of the stress induced by VIM-2 and SPM-1 in A. baumannii, further increasing its repertoire to resist the action of carbapenems.

### Bacterial strains and plasmids.

AB5075 and AB5075 Δ-*hns* were used in the present study. For expressing the different *bla* genes (*bla*_VIM-2_, *bla*_SPM-1_, and *bla*_NDM-1_) in A. baumannii, plasmid constructions of the MBL variants already containing *bla*_VIM-2_, *bla*_SPM-1,_ and *bla*_NDM-1_, as well as the empty vector pMBLe-OA (3), were used as a backbone to include the apramycin resistance gene (ArK^r^) to generate plasmids pMBLe-OA-ArK, pMBLe-VIM-2-ArK, pMBLe-SPM-1-ArK, and pMBLe-NDM-1-ArK, to be used in the multidrug-resistant (MDR) strains AB5075 and AB5075 Δ-*hns.* MBL expression was induced with low concentrations of IPTG (10 and 20 μM), as indicated.

### Electroporation.

Electrocompetent A. baumannii AB5075 and AB5075 Δ-*hns* cells were prepared as described previously (14). Briefly, isolated colonies of AB5075 and AB5075 Δ*-hns* were inoculated into 3 ml of LB broth and placed in a 37°C shaking incubator overnight. On the following day, 0.5 ml of the AB5075 and AB5075 Δ*-hns* cultures were transferred to separate conical tubes containing 50 ml LB broth prewarmed to 37°C. The tubes were incubated in a 37°C shaking incubator for 2 h to reach an optical density at 600 nm (OD_600_) of 0.3 to 0.5. The cells were then pelleted at 10,000 × *g* and washed twice with 25 ml of 10% glycerol at room temperature, and the pellet was resuspended in 1.5 ml of 10% glycerol. The electrocompetent cells were aliquoted and stored at –80°C.

A. baumannii AB5075 and AB5075 Δ-*hns* electrocompetent cells were mixed with 25 ng of plasmid DNA followed by electroporation with a Bio-Rad Gene Pulser instrument at 2.5 kV, 200 Ω, and 25 μF. The electroporated cells were placed in recovery with 1 ml of LB broth for 2 h at 37°C in a shaking incubator, followed by culturing overnight at 37°C on LB agar containing 15 μg/ml apramycin (15). At least 10 colonies were picked to confirm the presence of the different plasmids. To confirm their presence, plasmid extraction followed by gel electrophoresis analysis and PCR using the corresponding primers to amplify either *bla*_VIM-2_, *bla*_SPM-1,_ and *bla*_NDM-1_, and ArK (apramycin resistance gene) were performed.

### Growth curves.

Growth curves were conducted on 96-well plates in triplicate with strains AB5075 and AB5075 Δ-*hns* with pMBLe-OA-ArK, pMBLe-VIM-2-ArK, pMBLe-SPM-1-ArK, or pMBLe-NDM-1-ArK in LB plus 0, 10, or 20 μM IPTG and as much as 30 μg/ml apramycin. Overnight cultures were subcultured 1:50 in LB incubated for 15 h at 37°C with medium shaking. Growth was measured as the OD_600_ every 20 min using a Synergy 2 multimode plate reader (BioTek, Winooski, VT, USA) and Gen5 microplate reader software (BioTek).

### DNA-damaging agent susceptibility assays.

AB5075 and AB5075 Δ-*hns* cells were exposed to 0.2 μg/ml mitomycin C (MC), and a cell count was performed to measure cell killing as described previously (11). Assays were performed in triplicate, with at least three technical replicates per biological replicate. In addition, growth curves of strains AB5075 and AB5075 Δ-*hns* exposed to 0 or 8 μg/ml of levofloxacin (subinhibitory concentration) were performed as described above, and bacterial growth was measured every 20 min using a Synergy 2 multimode plate reader (BioTek, Winooski, VT, USA) and Gen5 microplate reader software (BioTek).

### Antibiotic susceptibility assays.

Imipenem (IMI) MICs at 0 and 10 μM IPTG were determined using liquid microdilution according to CLSI standards (16).
