# Supplementary figures and images for: The H-NS Regulator Plays a Role in the Stress Induced by Carbapenemase Expression in Acinetobacter baumannii
Source: mSphere. 2020 Aug 26;5(4):e00793-20. doi: 10.1128/mSphere.00793-20 (PMC7449629; doi:10.1128/mSphere.00793-20)

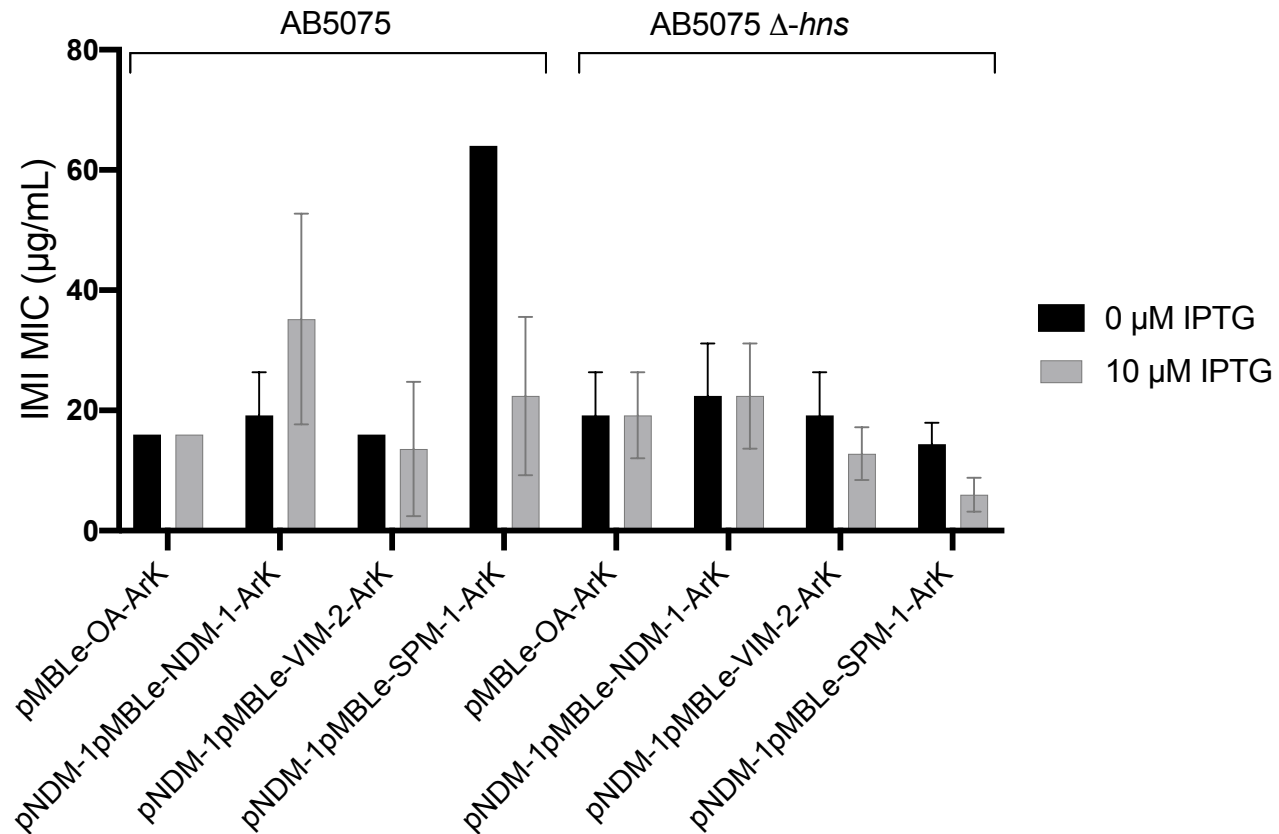

Supplement: FIG S1 [file mSphere.00793-20-sf001.pdf]
